# Usefulness of the Monti–Malone procedure as a reconstruction of the antegrade continence enema procedure: a case report

**DOI:** 10.1186/s40792-021-01197-5

**Published:** 2021-05-06

**Authors:** Koichi Saito, Yoshiaki Kinoshita, Yoshiaki Takahashi, Takashi Kobayashi, Yuhki Arai, Toshiyuki Ohyama, Naoki Yokota

**Affiliations:** Department of Pediatric Surgery, Niigata University Graduate School of Medical and Dental Sciences, 1-757 Asahimachi-dori, Chuo-ku, Niigata, Niigata Japan

**Keywords:** Antegrade continence enema, Monti–Malone procedure, Constipation

## Abstract

**Background:**

The antegrade continence enema (ACE) procedure is effective for severe constipation in patients with spina bifida and can improve quality of life (QOL). The Monti–Malone procedure (MM), which is a method of creating an enema tract from the colon, has been reported as an alternative to the ACE procedure when the appendix cannot be used. We report the usefulness of MM as a reconstruction of the antegrade continence enema procedure.

**Case presentation:**

Our patient was a 22-year-old man with congenital spina bifida and hydrocephalus. Ventriculoperitoneal (VP) shunt surgery was performed immediately after birth, and preventative appendectomy was carried out during VP shunt repair when 4 months old. At 5 years of age, the ACE procedure using a balloon-button gastrostomy tube was performed for intractable chronic constipation. Simple management was expected, but after 17 years of age, he experienced increased stool leakage around the gastrostomy tube and his QOL declined due to difficulty in managing the ACE. Therefore, reconstruction of the ACE procedure by MM was performed. After reconstruction, the ACE performed well without any complications. The patient is currently satisfied because management of the ACE is easier than before, and his QOL has markedly improved without stool leakage and dermatitis.

**Conclusions:**

MM is less likely to cause complications and is useful as a reconstruction of the ACE procedure.

## Background

The antegrade continence enema (ACE) procedure is effective for severe constipation in patients with spina bifida and can improve quality of life (QOL). The Malone ACE procedure [1] is a method of constructing an ACE using the appendix, and is widely used. In cases in which the appendix is not available for ACE, the Monti–Malone procedure (MM) has been reported [2]. We report the usefulness of the Monti–Malone procedure as a reconstruction of the antegrade continence enema procedure.

## Case presentation

**Fig. 1:**
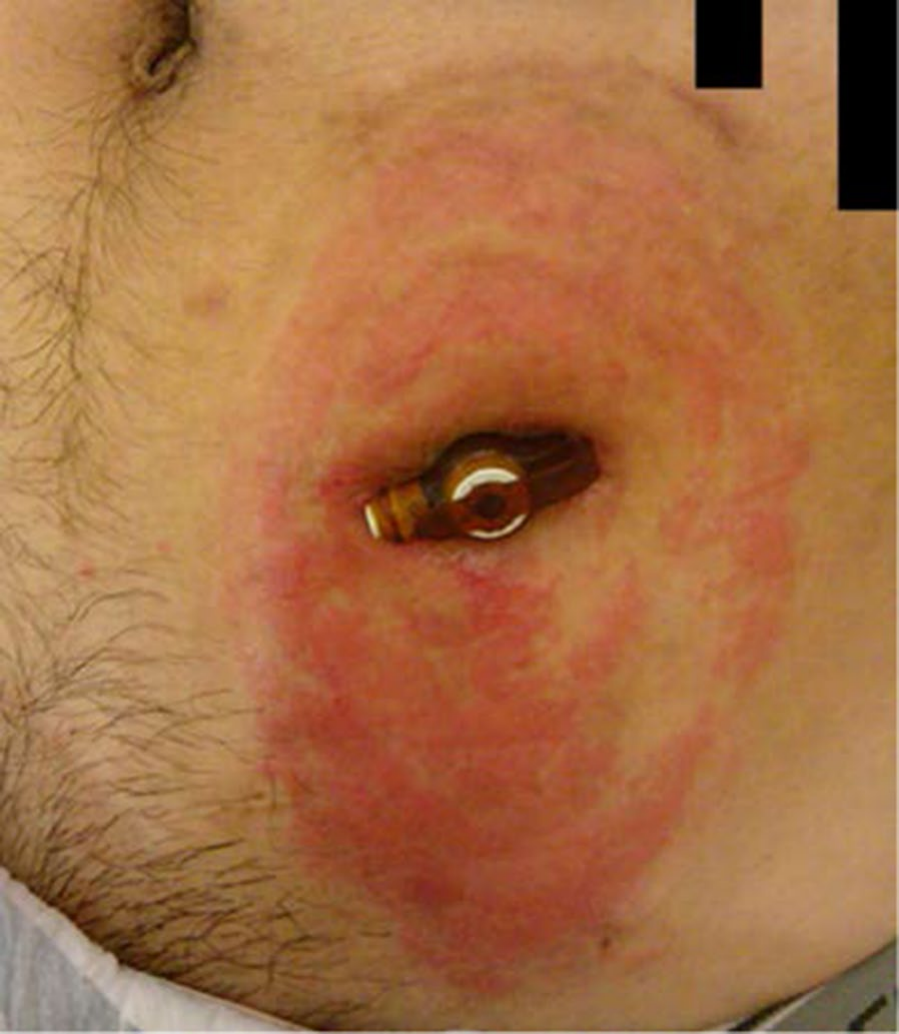
The ACE procedure using a balloon-button gastrostomy tube and skin findings just before reconstruction. Dermatitis was observed around the gastrostomy tube

The patient was a 22-year-old man with spina bifida and hydrocephalus since birth, with paralysis of the lower limbs. Ventriculoperitoneal (VP) shunt surgery was performed immediately after birth, and appendectomy was carried out preventively during VP shunt repair at 4 months old. At 5 years of age, outpatient follow-up was started for chronic constipation due to spina bifida. An ACE procedure using a balloon-button gastrostomy tube was constructed in the lower left abdomen for simple management of defecation, and was thus managed for more than 10 years. However, stool leakage around the gastrostomy tube increased from the age of 17 years, and dermatitis began to appear (Fig. 1). Therefore, the gastrostomy tube was changed to one of appropriate size and shaft length, and covered with a stoma pouch to reduce skin irritation. However, his QOL declined due to the need for frequent pouch replacement. Thus, we decided to reconstruct the ACE procedure again using MM.

**Fig. 2:**
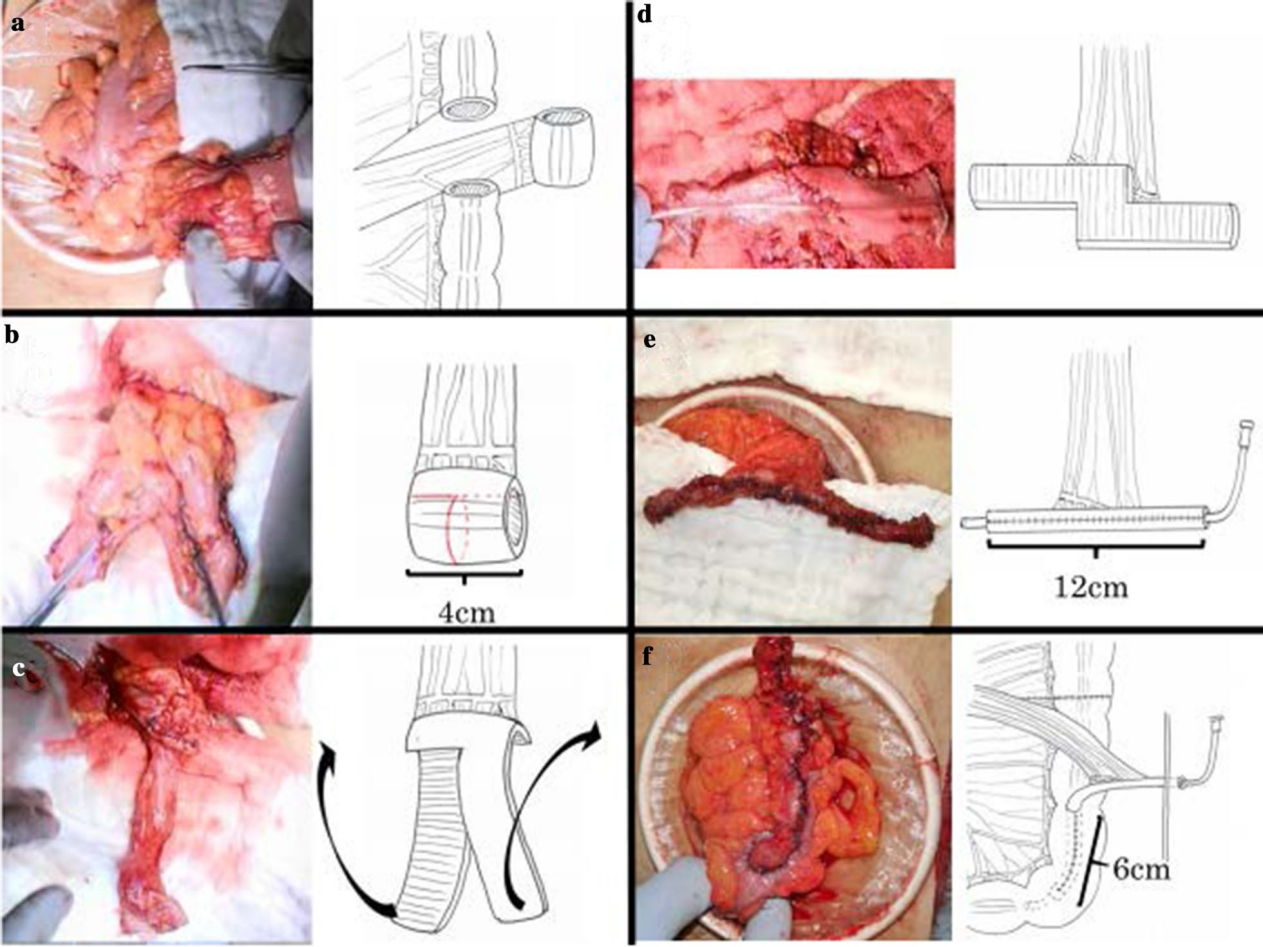
Monti–Malone procedure. **a** A 4-cm section of colon was secured while preserving the feeding blood vessels. **b**–**d** A two-thirds cut was made in the center of the free intestinal tract. Each left and right intestine was incised longitudinally and widened in a reed-shaped manner. **e** A 12-cm intestinal tube was created by suturing longitudinally. **f** The intestinal tube and colon were subjected to end-to-side anastomosis. We used Witzel’s method for coverage of the intestinal tube. The length of that coverage was 6 cm

After sufficient intestinal preparation, MM was performed (Fig. 2). The part of the sigmoid colon where the gastrostomy tube had been inserted was resected, and a sufficiently long intestinal tract from the descending colon to the sigmoid colon was released. The mesentery was dissected while preserving feeding blood vessels to create a free intestinal tract. The center of the free intestinal tract was cut two-thirds of its width, and the left and right intestines were each incised longitudinally and widened in a reed shape. A 12-cm intestinal tube was created by longitudinal suturing with reference to the literature [3], and an 8 Fr tube (Atom Multipurpose Tube™, Atom Medical, Tokyo) was used as a stent. The intestinal tube and colon were subjected to end-to-side anastomosis, and covered by Witzel’s method 6 cm from the anastomosis of the intestinal tube. An exit for the intestinal tube was created in the left abdomen, and the intestinal tube was pulled out and sutured to the skin.

**Fig. 3:**
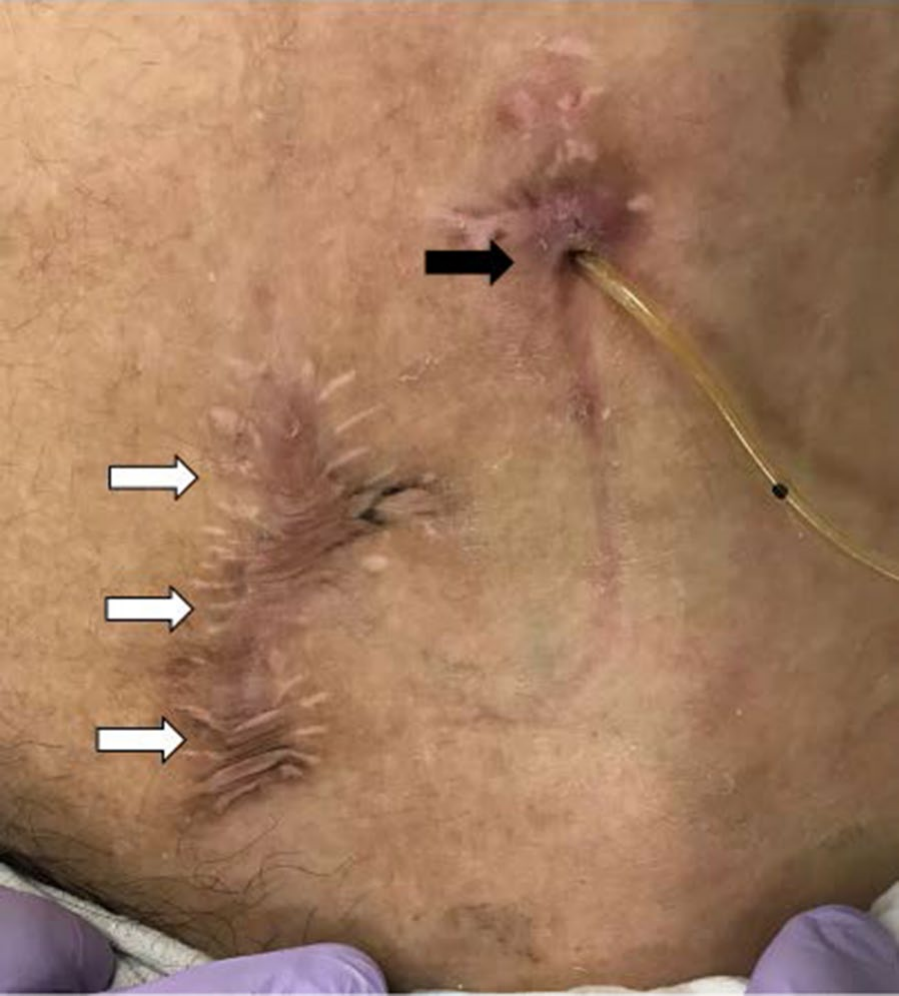
Appearance one and a half years after the operation. The exit for intestinal tube (black arrow) and the wound after closure of the ACE procedure that used a balloon-button gastrostomy tube (white arrow)

On the 21st day after the operation, ACE from the intestinal tube was started. ACE could be performed without any problems, and the patient was discharged on the 29th day after surgery. Currently, approximately one and a half years have passed since the operation. The intestinal tube is only covered with gauze, which makes management easier than before. The QOL markedly improved without stool leakage and dermatitis (Fig. 3), and the patient is satisfied.

## Discussion

ACE is widely recognized for its use in severe constipation. In 1990, Malone et al. reported the ACE procedure by inverting the appendix and using it as a catheter introduction route [1]. Since then, the Malone procedure has been widely used and modified for treating severe constipation. In 1997, Monti et al. reported a method by which a conduit could be formed from the ileum as a method of urinary diversion, in those cases where the appendix could not be used [4]. In 2002, MM was also applied to the descending or sigmoid colon by creating and anastomosing the conduit [2].

In spina bifida, the efficacy rate of ACE is reported to be 84% [5], and patient satisfaction is reported to be 89%-98% [6]. Currently, transanal irrigation systems are spreading. The ACE procedure, which includes MM, might be applied in cases where the transanal irrigation system is not effective. The advantages of MM are the more precise flow of the enema solution into the colon and the ability to perform enemas on the oral side of the colon that cannot be reached with a transanal irrigation system depending on the construction position. Furthermore, for patients with lower limb paralysis, such as this case, MM could keep the management simpler because of fewer items. For constipation with a neurological cause, the etiology is often in the rectosigmoid region, and ACE to the descending or sigmoid colon is said to be physiologically suitable [7], and requires less time and amount of injection for a single enema. Therefore, we think the MM should be indicated in cases without the appendix because the original Malone procedure is less invasive; however, it is better to construct a MM procedure in the descending colon or sigmoid colon if the appendix cannot be used.

The ACE procedure using a balloon-button gastrostomy tube is a simple and safe method of construction. However, granulation and stool leakage around the gastrostomy tube have been reported as the main complications [8, 9]. This ACE procedure is constructed by directly suturing to the colon and skin as in a gastrostomy; therefore, stool leakage is likely to occur. Complications can be minimized by using the appropriate size of gastrostomy tube in each patient [9]. In this case, the ACE procedure using a balloon-button gastrostomy tube was initially constructed in anticipation of simple surgical technique and management. However, stool leakage increased and dermatitis developed, despite changing the gastrostomy tube to one with appropriate size and shaft length and using a stoma pouch. Although there have been no reports on long-term follow-up of cases using gastrostomy tubes, it is thought that stool leakage and dermatitis would be a late complication in such cases.

Intestinal ischemia is a concern for MM; however, avoiding tension and preserving at least two or more feeding vessels when creating an intestinal tube is an effective preventative measure [10]. MM makes it easy to insert the stent tube, and stool leakage is unlikely to occur because of the distance between the colon and the skin [11]. Reconstruction of the ACE procedure by MM not only eliminates stool leakage, but also simplifies management and improves the patient's QOL.

## Conclusions

The ACE procedure remains essential for defecation management, and it is important to ensure that simple management can be performed and no complications occur. MM is less likely to cause complications and is useful as a reconstruction for the ACE procedure.

## Data Availability

Not applicable.

## Abbreviations

ACE: Antegrade continence enema
QOL: Quality of life
MM: Monti–Malone procedure
VP: Ventriculoperitoneal
